# Supplementary material for: A Dominant-Negative Mutant of ANXA7 Impairs Calcium Signaling and Enhances the Proliferation of Prostate Cancer Cells by Downregulating the IP3 Receptor and the PI3K/mTOR Pathway
Source: Int J Mol Sci. 2023 May 16;24(10):8818. doi: 10.3390/ijms24108818 (PMC10219041; doi:10.3390/ijms24108818)
Supplement: Supplementary file 1 [file ijms-24-08818-s001.zip › ijms-2339173-supplementary.pdf]

Supplementary Data

Table S1: *ANXA7 cellular clustering with neighboring genes:* The RNA data was used to cluster genes according to their expression across samples. The resulting clusters have been manually annotated to describe common features in terms of function and specificity. The annotation of the cluster is displayed together with a confidence score of the gene's assignment to the cluster. The confidence is calculated as the fraction of times the gene was assigned to this cluster in repeated calculations and is reported between 0 to 1, where 1 is the highest possible confidence. Table shows the 15 most similar genes in terms of expression profile. Data analyzed from the publicly available data from Human Protein Atlas and Genotype-Tissue Expression (GTEx) project.

Neighbour	Description	Correlation	Cluster
MRPL39	Mitochondrial ribosomal protein L39	0.8982	8
RABL3	RAB, member of RAS oncogene family like 3	0.8982	8
DENR	Density regulated re-initiation and release factor	0.8842	8
RRM2B	Ribonucleotide reductase regulatory TP53 inducible subunit M2B	0.8737	8
HNRNPH2	Heterogeneous nuclear ribonucleoprotein H2	0.8702	48
TSNAX	Translin associated factor X	0.8667	8
IARS2	Isoleucyl-tRNA synthetase 2, mitochondrial	0.8596	8
APOOL	Apolipoprotein O like	0.8579	8
ATPAF2	ATP synthase mitochondrial F1 complex assembly factor 2	0.8544	8
RNF14	Ring finger protein 14	0.8491	8
CISD1	CDGSH iron sulfur domain 1	0.8474	8
PRDX3	Peroxiredoxin 3	0.8456	8
OPA3	Outer mitochondrial membrane lipid metabolism regulator OPA3	0.8439	8
TRAPPC13	Trafficking protein particle complex 13	0.8368	8
EI24	EI24 autophagy associated transmembrane protein	0.8368	48

Supplementary Figures

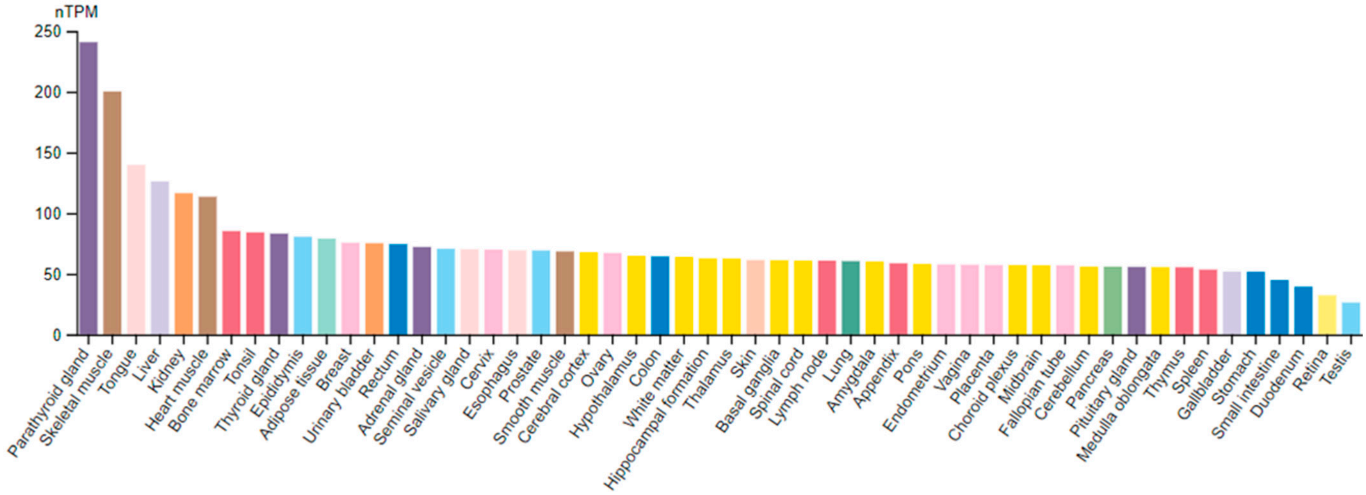

Figure S1: Organ/tissue-based RNA expression of ANXA7. The consensus dataset consists of normalized expression (nTPM) levels for 55 tissue types, created by combining the HPA and GTEx transcriptomics datasets using the internal normalization pipeline. Color-coding is based on tissue groups, each consisting of tissues with functional features in common. Date obtained from The human protein atlas (https://www.proteinatlas.org).

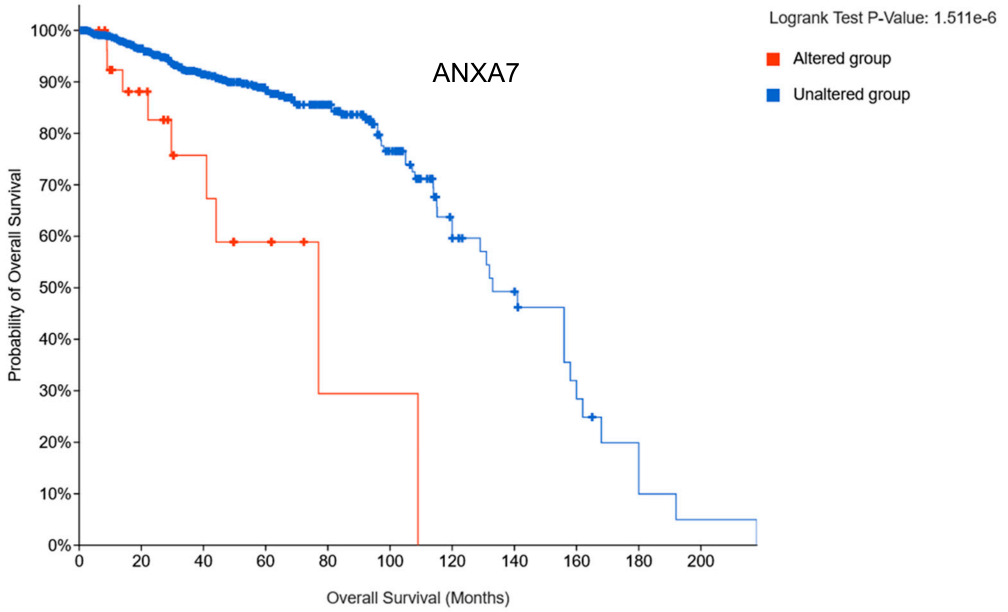

	Number of Cases, Total	Number of Events	Median Months Overall (95% CI)
Altered group	**29**	**9**	**77.00 (41.00 - NA)**
Unaltered group	**1446**	**143**	**133.00 (120.00 - 160.00)**

Figure S2: Prostate cancer survival data in terms of ANXA7 Copy number alteration (CNA) Vs un-altered in tumor samples from patients' samples. The data was generated from a combined Study (n=10999; samples) Querying 10999 samples / 10580 patients in 26 studies using cBioportal platform. Gene name: ANXA7.
